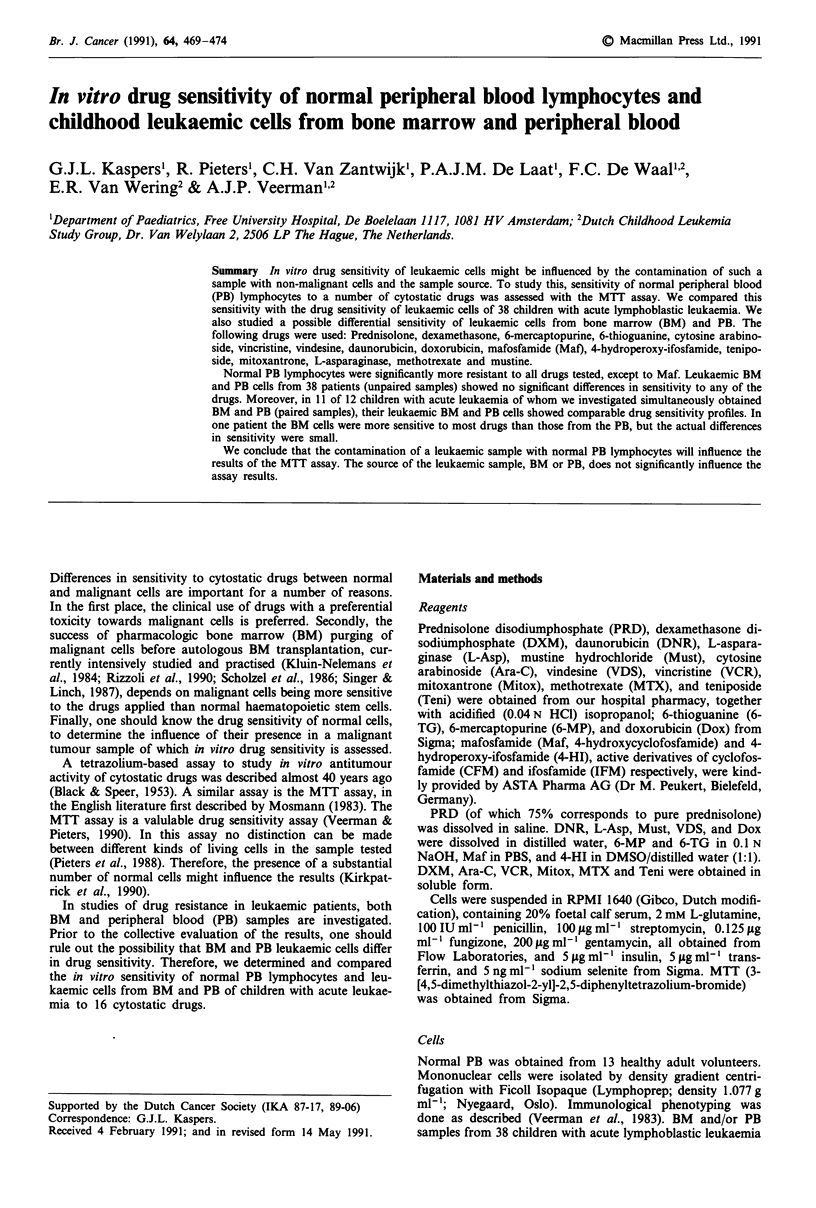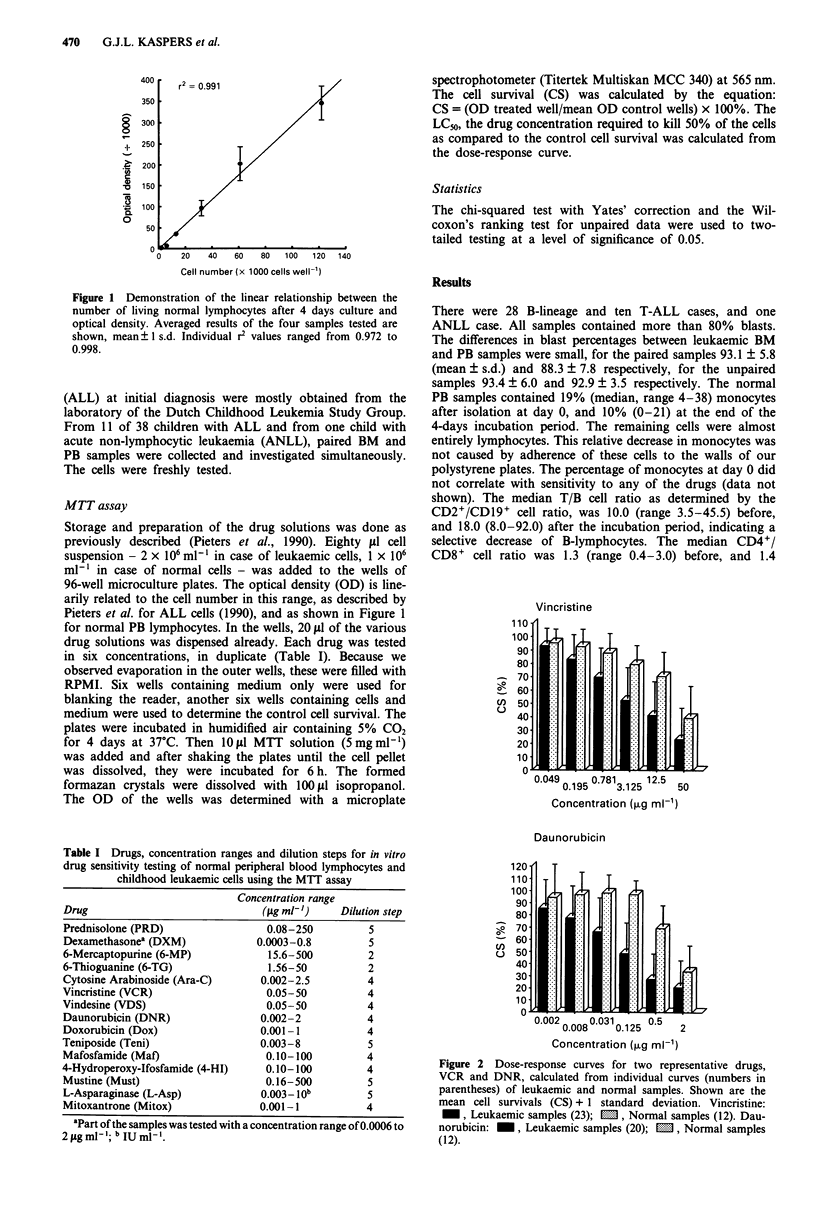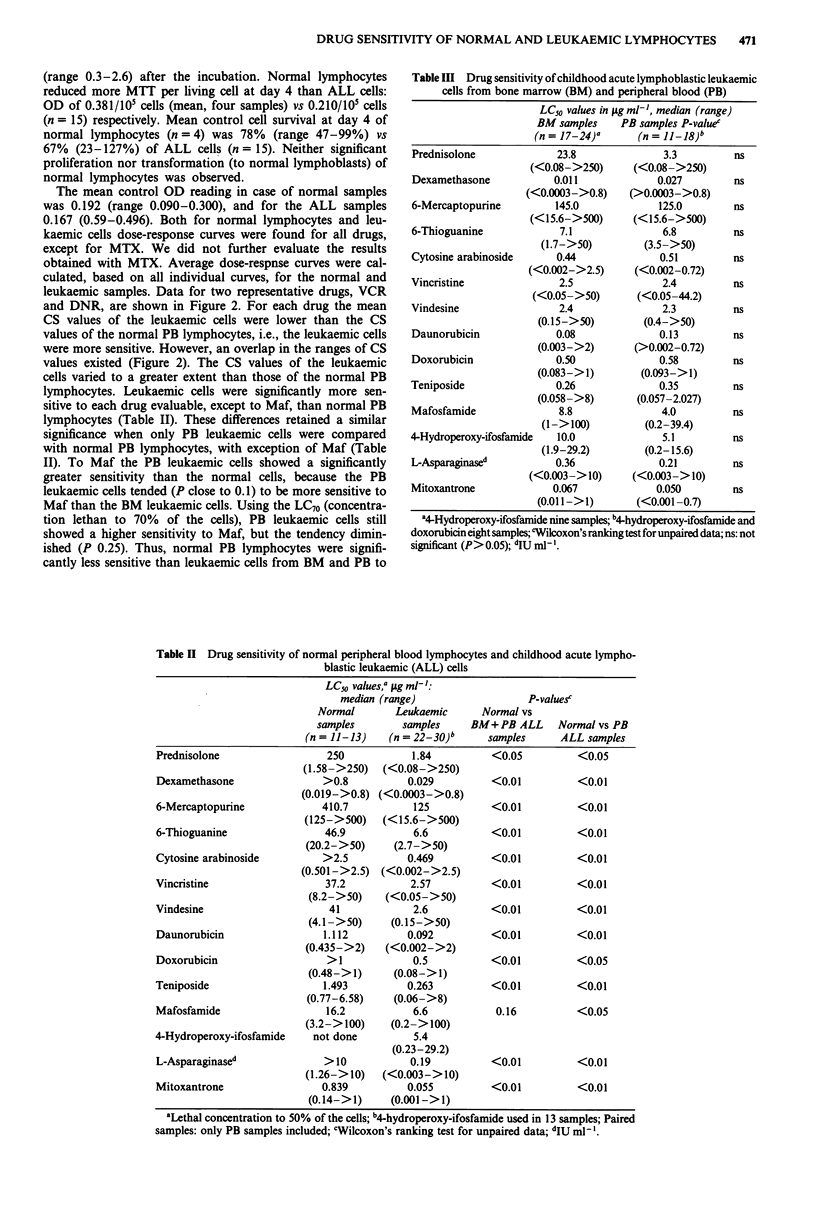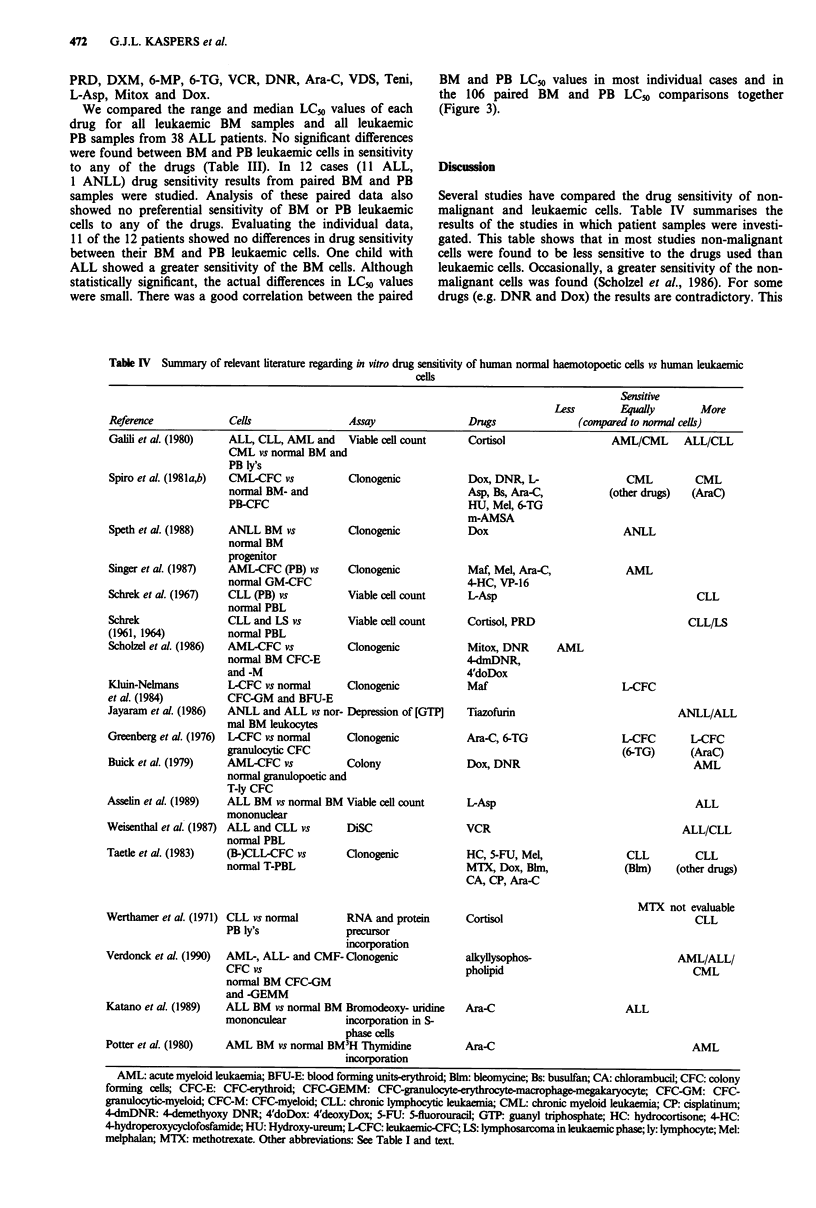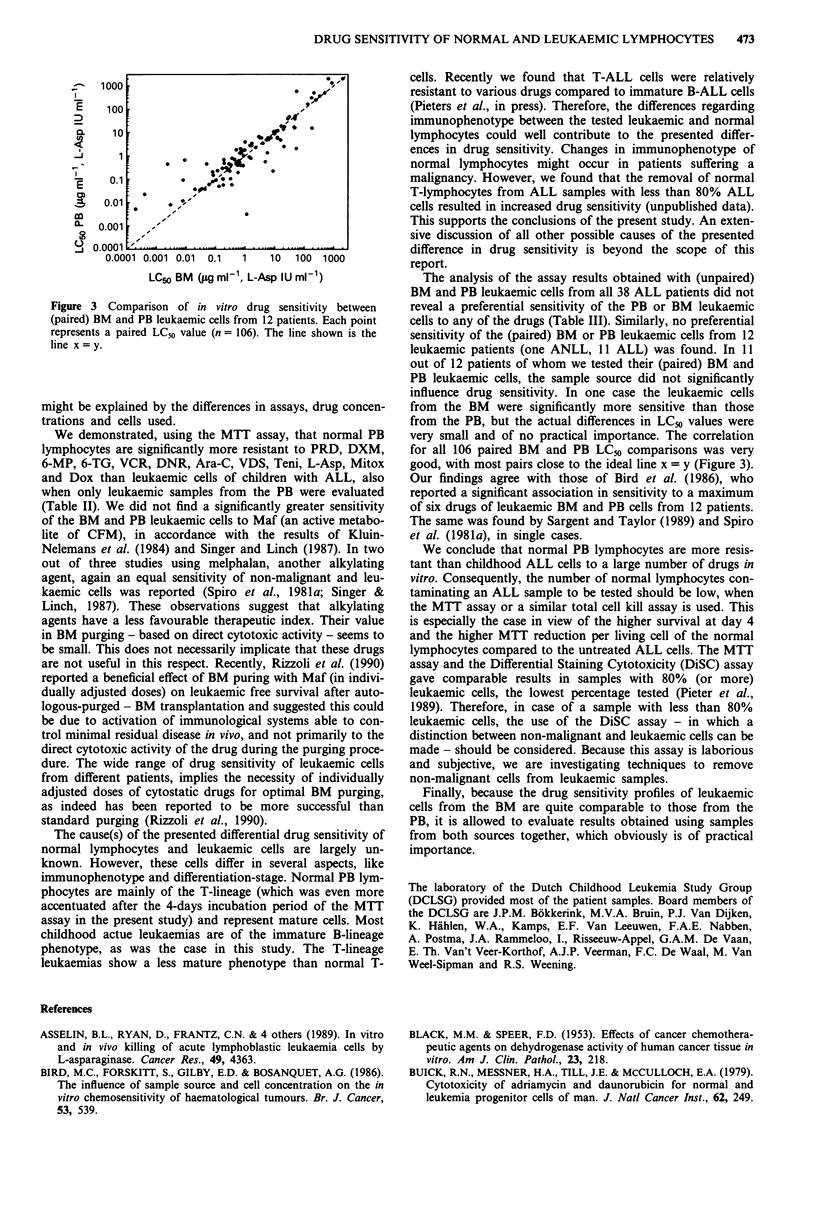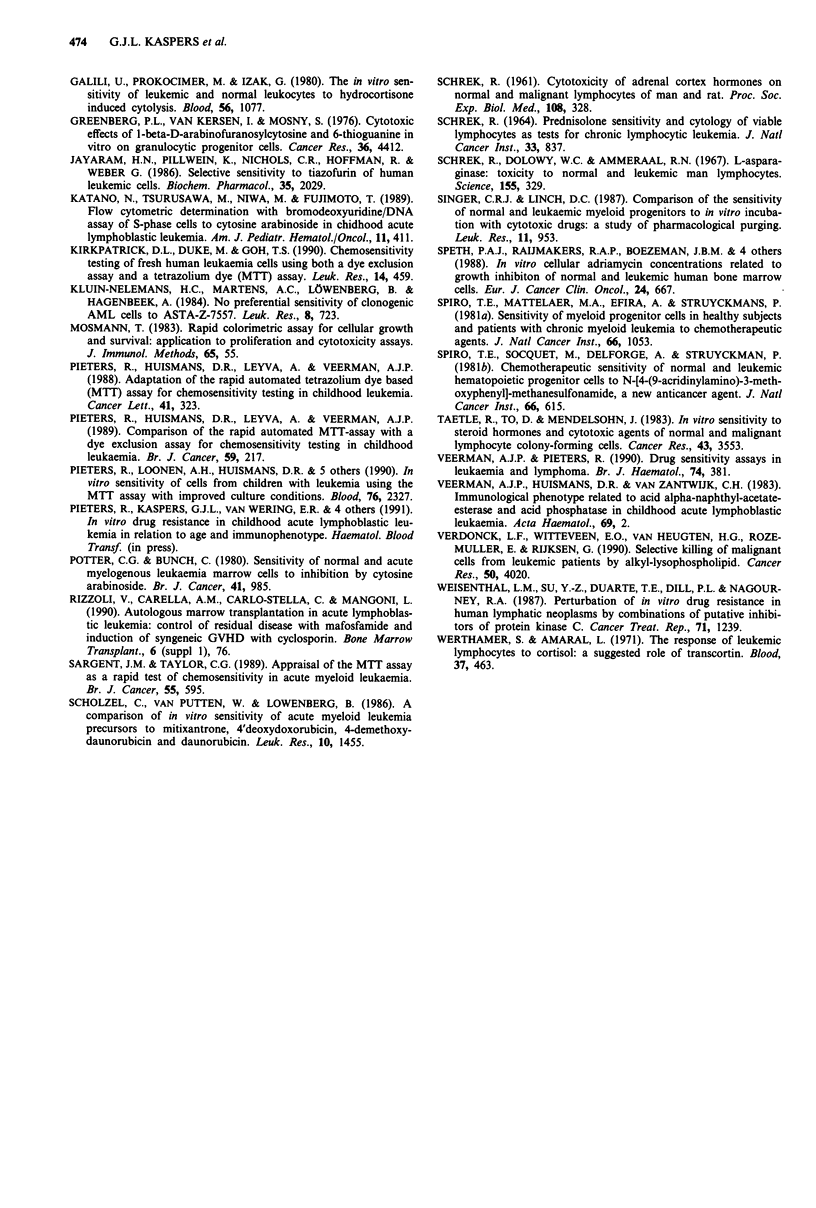# In vitro drug sensitivity of normal peripheral blood lymphocytes and childhood leukaemic cells from bone marrow and peripheral blood.

**DOI:** 10.1038/bjc.1991.333

**Published:** 1991-09

**Authors:** G. J. Kaspers, R. Pieters, C. H. Van Zantwijk, P. A. De Laat, F. C. De Waal, E. R. Van Wering, A. J. Veerman

**Affiliations:** Department of Paediatrics, Free University Hospital, Amsterdam, The Netherlands.

## Abstract

In vitro drug sensitivity of leukaemic cells might be influenced by the contamination of such a sample with non-malignant cells and the sample source. To study this, sensitivity of normal peripheral blood (PB) lymphocytes to a number of cytostatic drugs was assessed with the MTT assay. We compared this sensitivity with the drug sensitivity of leukaemic cells of 38 children with acute lymphoblastic leukaemia. We also studied a possible differential sensitivity of leukaemic cells from bone marrow (BM) and PB. The following drugs were used: Prednisolone, dexamethasone, 6-mercaptopurine, 6-thioguanine, cytosine arabinoside, vincristine, vindesine, daunorubicin, doxorubicin, mafosfamide (Maf), 4-hydroperoxy-ifosfamide, teniposide, mitoxantrone, L-asparaginase, methotrexate and mustine. Normal PB lymphocytes were significantly more resistant to all drugs tested, except to Maf. Leukaemic BM and PB cells from 38 patients (unpaired samples) showed no significant differences in sensitivity to any of the drugs. Moreover, in 11 of 12 children with acute leukaemia of whom we investigated simultaneously obtained BM and PB (paired samples), their leukaemic BM and PB cells showed comparable drug sensitivity profiles. In one patient the BM cells were more sensitive to most drugs than those from the PB, but the actual differences in sensitivity were small. We conclude that the contamination of a leukaemic sample with normal PB lymphocytes will influence the results of the MTT assay. The source of the leukaemic sample, BM or PB, does not significantly influence the assay results.


Br. J. Cancer (1991), 64, 469-474 © Macmillan Press Ltd., 1991

### *In vitro* drug sensitivity of normal peripheral blood lymphocytes and childhood leukaemic cells from bone marrow and peripheral blood

G.J.L. Kaspers[1], R. Pieters[1], C.H. Van Zantwijk[1], P.A.J.M. De Laat[1], F.C. De Waal[1,2], E.R. Van Wering[2] & A.J.P. Veerman[1,2]

[1]*Department of Paediatrics, Free University Hospital, De Boelelaan 1117, 1081 HV Amsterdam;* [2]*Dutch Childhood Leukemia Study Group, Dr. Van Welylaan 2, 2506 LP The Hague, The Netherlands.*

**Summary** *In vitro* drug sensitivity of leukaemic cells might be influenced by the contamination of such a sample with non-malignant cells and the sample source. To study this, sensitivity of normal peripheral blood (PB) lymphocytes to a number of cytostatic drugs was assessed with the MTT assay. We compared this sensitivity with the drug sensitivity of leukaemic cells of 38 children with acute lymphoblastic leukaemia. We also studied a possible differential sensitivity of leukaemic cells from bone marrow (BM) and PB. The following drugs were used: Prednisolone, dexamethasone, 6-mercaptopurine, 6-thioguanine, cytosine arabinoside, vincristine, vindesine, daunorubicin, doxorubicin, mafosfamide (Maf), 4-hydroperoxy-ifosfamide, teniposide, mitoxantrone, L-asparaginase, methotrexate and mustine.

Normal PB lymphocytes were significantly more resistant to all drugs tested, except to Maf. Leukaemic BM and PB cells from 38 patients (unpaired samples) showed no significant differences in sensitivity to any of the drugs. Moreover, in 11 of 12 children with acute leukaemia of whom we investigated simultaneously obtained BM and PB (paired samples), their leukaemic BM and PB cells showed comparable drug sensitivity profiles. In one patient the BM cells were more sensitive to most drugs than those from the PB, but the actual differences in sensitivity were small.

We conclude that the contamination of a leukaemic sample with normal PB lymphocytes will influence the results of the MTT assay. The source of the leukaemic sample, BM or PB, does not significantly influence the assay results.

Differences in sensitivity to cytostatic drugs between normal and malignant cells are important for a number of reasons. In the first place, the clinical use of drugs with a preferential toxicity towards malignant cells is preferred. Secondly, the success of pharmacologic bone marrow (BM) purging of malignant cells before autologous BM transplantation, currently intensively studied and practised (Kluin-Nelemans *et al.*, 1984; Rizzoli *et al.*, 1990; Scholzel *et al.*, 1986; Singer & Linch, 1987), depends on malignant cells being more sensitive to the drugs applied than normal haematopoietic stem cells. Finally, one should know the drug sensitivity of normal cells, to determine the influence of their presence in a malignant tumour sample of which *in vitro* drug sensitivity is assessed.

A tetrazolium-based assay to study *in vitro* antitumour activity of cytostatic drugs was described almost 40 years ago (Black & Speer, 1953). A similar assay is the MTT assay, in the English literature first described by Mosmann (1983). The MTT assay is a valulable drug sensitivity assay (Veerman & Pieters, 1990). In this assay no distinction can be made between different kinds of living cells in the sample tested (Pieters *et al.*, 1988). Therefore, the presence of a substantial number of normal cells might influence the results (Kirkpatrick *et al.*, 1990).

In studies of drug resistance in leukaemic patients, both BM and peripheral blood (PB) samples are investigated. Prior to the collective evaluation of the results, one should rule out the possibility that BM and PB leukaemic cells differ in drug sensitivity. Therefore, we determined and compared the *in vitro* sensitivity of normal PB lymphocytes and leukaemic cells from BM and PB of children with acute leukaemia to 16 cytostatic drugs.

Supported by the Dutch Cancer Society (IKA 87-17, 89-06)
Correspondence: G.J.L. Kaspers.
Received 4 February 1991; and in revised form 14 May 1991.

### Materials and methods

### *Reagents*

Prednisolone disodiumphosphate (PRD), dexamethasone disodiumphosphate (DXM), daunorubicin (DNR), L-asparaginase (L-Asp), mustine hydrochloride (Must), cytosine arabinoside (Ara-C), vindesine (VDS), vincristine (VCR), mitoxantrone (Mitox), methotrexate (MTX), and teniposide (Teni) were obtained from our hospital pharmacy, together with acidified (0.04 N HCl) isopropanol; 6-thioguanine (6-TG), 6-mercaptopurine (6-MP), and doxorubicin (Dox) from Sigma; mafosfamide (Maf, 4-hydroxycyclofosfamide) and 4-hydroperoxy-ifosfamide (4-HI), active derivatives of cyclofosfamide (CFM) and ifosfamide (IFM) respectively, were kindly provided by ASTA Pharma AG (Dr M. Peukert, Bielefeld, Germany).

PRD (of which 75% corresponds to pure prednisolone) was dissolved in saline. DNR, L-Asp, Must, VDS, and Dox were dissolved in distilled water, 6-MP and 6-TG in 0.1 N NaOH, Maf in PBS, and 4-HI in DMSO/distilled water (1:1). DXM, Ara-C, VCR, Mitox, MTX and Teni were obtained in soluble form.

Cells were suspended in RPMI 1640 (Gibco, Dutch modification), containing 20% foetal calf serum, 2 mM L-glutamine, 100 IU $ml^{-1}$ penicillin, 100 $\mu g\ ml^{-1}$ streptomycin, 0.125 $\mu g\ ml^{-1}$ fungizone, 200 $\mu g\ ml^{-1}$ gentamycin, all obtained from Flow Laboratories, and 5 $\mu g\ ml^{-1}$ insulin, 5 $\mu g\ ml^{-1}$ transferrin, and 5 $ng\ ml^{-1}$ sodium selenite from Sigma. MTT (3-[4,5-dimethylthiazol-2-yl]-2,5-diphenyltetrazolium-bromide) was obtained from Sigma.

### *Cells*

Normal PB was obtained from 13 healthy adult volunteers. Mononuclear cells were isolated by density gradient centrifugation with Ficoll Isopaque (Lymphoprep; density 1.077 g $ml^{-1}$; Nyegaard, Oslo). Immunological phenotyping was done as described (Veerman *et al.*, 1983). BM and/or PB samples from 38 children with acute lymphoblastic leukaemia

**Figure 1** Demonstration of the linear relationship between the number of living normal lymphocytes after 4 days culture and optical density. Averaged results of the four samples tested are shown, mean ± 1 s.d. Individual $r^2$ values ranged from 0.972 to 0.998.

(ALL) at initial diagnosis were mostly obtained from the laboratory of the Dutch Childhood Leukemia Study Group. From 11 of 38 children with ALL and from one child with acute non-lymphocytic leukaemia (ANLL), paired BM and PB samples were collected and investigated simultaneously. The cells were freshly tested.

### *MTT assay*

Storage and preparation of the drug solutions was done as previously described (Pieters *et al.*, 1990). Eighty μl cell suspension – $2 \times 10^6$ ml$^{-1}$ in case of leukaemic cells, $1 \times 10^6$ ml$^{-1}$ in case of normal cells – was added to the wells of 96-well microculture plates. The optical density (OD) is linearily related to the cell number in this range, as described by Pieters *et al.* for ALL cells (1990), and as shown in Figure 1 for normal PB lymphocytes. In the wells, 20 μl of the various drug solutions was dispensed already. Each drug was tested in six concentrations, in duplicate (Table I). Because we observed evaporation in the outer wells, these were filled with RPMI. Six wells containing medium only were used for blanking the reader, another six wells containing cells and medium were used to determine the control cell survival. The plates were incubated in humidified air containing 5% $CO_2$ for 4 days at 37°C. Then 10 μl MTT solution (5 mg ml$^{-1}$) was added and after shaking the plates until the cell pellet was dissolved, they were incubated for 6 h. The formed formazan crystals were dissolved with 100 μl isopropanol. The OD of the wells was determined with a microplate spectrophotometer (Titertek Multiskan MCC 340) at 565 nm. The cell survival (CS) was calculated by the equation: CS = (OD treated well/mean OD control wells) × 100%. The $LC_{50}$, the drug concentration required to kill 50% of the cells as compared to the control cell survival was calculated from the dose-response curve.

### *Statistics*

The chi-squared test with Yates' correction and the Wilcoxon's ranking test for unpaired data were used to two-tailed testing at a level of significance of 0.05.

### Results

There were 28 B-lineage and ten T-ALL cases, and one ANLL case. All samples contained more than 80% blasts. The differences in blast percentages between leukaemic BM and PB samples were small, for the paired samples 93.1 ± 5.8 (mean ± s.d.) and 88.3 ± 7.8 respectively, for the unpaired samples 93.4 ± 6.0 and 92.9 ± 3.5 respectively. The normal PB samples contained 19% (median, range 4–38) monocytes after isolation at day 0, and 10% (0–21) at the end of the 4-days incubation period. The remaining cells were almost entirely lymphocytes. This relative decrease in monocytes was not caused by adherence of these cells to the walls of our polystyrene plates. The percentage of monocytes at day 0 did not correlate with sensitivity to any of the drugs (data not shown). The median T/B cell ratio as determined by the $CD2^+/CD19^+$ cell ratio, was 10.0 (range 3.5–45.5) before, and 18.0 (8.0–92.0) after the incubation period, indicating a selective decrease of B-lymphocytes. The median $CD4^+/CD8^+$ cell ratio was 1.3 (range 0.4–3.0) before, and 1.4

**Table I** Drugs, concentration ranges and dilution steps for *in vitro* drug sensitivity testing of normal peripheral blood lymphocytes and childhood leukaemic cells using the MTT assay

| *Drug* | *Concentration range (μg ml⁻¹)* | *Dilution step* |
|---|---|---|
| Prednisolone (PRD) | 0.08–250 | 5 |
| Dexamethasone[a] (DXM) | 0.0003–0.8 | 5 |
| 6-Mercaptopurine (6-MP) | 15.6–500 | 2 |
| 6-Thioguanine (6-TG) | 1.56–50 | 2 |
| Cytosine Arabinoside (Ara-C) | 0.002–2.5 | 4 |
| Vincristine (VCR) | 0.05–50 | 4 |
| Vindesine (VDS) | 0.05–50 | 4 |
| Daunorubicin (DNR) | 0.002–2 | 4 |
| Doxorubicin (Dox) | 0.001–1 | 4 |
| Teniposide (Teni) | 0.003–8 | 5 |
| Mafosfamide (Maf) | 0.10–100 | 4 |
| 4-Hydroperoxy-Ifosfamide (4-HI) | 0.10–100 | 4 |
| Mustine (Must) | 0.16–500 | 5 |
| L-Asparaginase (L-Asp) | 0.003–10[b] | 5 |
| Mitoxantrone (Mitox) | 0.001–1 | 4 |

[a]Part of the samples was tested with a concentration range of 0.0006 to 2 μg ml$^{-1}$; [b] IU ml$^{-1}$.

**Figure 2** Dose-response curves for two representative drugs, VCR and DNR, calculated from individual curves (numbers in parentheses) of leukaemic and normal samples. Shown are the mean cell survivals (CS) + 1 standard deviation. Vincristine: ■, Leukaemic samples (23); ▒, Normal samples (12). Daunorubicin: ■, Leukaemic samples (20); ▒, Normal samples (12).

(range 0.3–2.6) after the incubation. Normal lymphocytes reduced more MTT per living cell at day 4 than ALL cells: OD of 0.381/$10^5$ cells (mean, four samples) *vs* 0.210/$10^5$ cells ($n = 15$) respectively. Mean control cell survival at day 4 of normal lymphocytes ($n = 4$) was 78% (range 47–99%) *vs* 67% (23–127%) of ALL cells ($n = 15$). Neither significant proliferation nor transformation (to normal lymphoblasts) of normal lymphocytes was observed.

The mean control OD reading in case of normal samples was 0.192 (range 0.090–0.300), and for the ALL samples 0.167 (0.59–0.496). Both for normal lymphocytes and leukaemic cells dose-response curves were found for all drugs, except for MTX. We did not further evaluate the results obtained with MTX. Average dose-respnse curves were calculated, based on all individual curves, for the normal and leukaemic samples. Data for two representative drugs, VCR and DNR, are shown in Figure 2. For each drug the mean CS values of the leukaemic cells were lower than the CS values of the normal PB lymphocytes, i.e., the leukaemic cells were more sensitive. However, an overlap in the ranges of CS values existed (Figure 2). The CS values of the leukaemic cells varied to a greater extent than those of the normal PB lymphocytes. Leukaemic cells were significantly more sensitive to each drug evaluable, except to Maf, than normal PB lymphocytes (Table II). These differences retained a similar significance when only PB leukaemic cells were compared with normal PB lymphocytes, with exception of Maf (Table II). To Maf the PB leukaemic cells showed a significantly greater sensitivity than the normal cells, because the PB leukaemic cells tended ($P$ close to 0.1) to be more sensitive to Maf than the BM leukaemic cells. Using the $LC_{70}$ (concentration lethan to 70% of the cells), PB leukaemic cells still showed a higher sensitivity to Maf, but the tendency diminished ($P$ 0.25). Thus, normal PB lymphocytes were significantly less sensitive than leukaemic cells from BM and PB to

**Table III** Drug sensitivity of childhood acute lymphoblastic leukaemic cells from bone marrow (BM) and peripheral blood (PB)

| | *$LC_{50}$ values in µg $ml^{-1}$, median (range)* BM samples ($n = 17–24$)[a] | PB samples ($n = 11–18$)[b] | P-value[c] |
|---|---|---|---|
| Prednisolone | 23.8 (<0.08–>250) | 3.3 (<0.08–>250) | ns |
| Dexamethasone | 0.011 (<0.0003–>0.8) | 0.027 (>0.0003–>0.8) | ns |
| 6-Mercaptopurine | 145.0 (<15.6–>500) | 125.0 (<15.6–>500) | ns |
| 6-Thioguanine | 7.1 (1.7–>50) | 6.8 (3.5–>50) | ns |
| Cytosine arabinoside | 0.44 (<0.002–>2.5) | 0.51 (<0.002–0.72) | ns |
| Vincristine | 2.5 (<0.05–>50) | 2.4 (<0.05–44.2) | ns |
| Vindesine | 2.4 (0.15–>50) | 2.3 (0.4–>50) | ns |
| Daunorubicin | 0.08 (0.003–>2) | 0.13 (>0.002–0.72) | ns |
| Doxorubicin | 0.50 (0.083–>1) | 0.58 (0.093–>1) | ns |
| Teniposide | 0.26 (0.058–>8) | 0.35 (0.057–2.027) | ns |
| Mafosfamide | 8.8 (1–>100) | 4.0 (0.2–39.4) | ns |
| 4-Hydroperoxy-ifosfamide | 10.0 (1.9–29.2) | 5.1 (0.2–15.6) | ns |
| L-Asparaginase[d] | 0.36 (<0.003–>10) | 0.21 (<0.003–>10) | ns |
| Mitoxantrone | 0.067 (0.011–>1) | 0.050 (<0.001–0.7) | ns |

[a]4-Hydroperoxy-ifosfamide nine samples; [b]4-hydroperoxy-ifosfamide and doxorubicin eight samples; [c]Wilcoxon's ranking test for unpaired data; ns: not significant ($P > 0.05$); [d]IU $ml^{-1}$.

**Table II** Drug sensitivity of normal peripheral blood lymphocytes and childhood acute lymphoblastic leukaemic (ALL) cells

| | *$LC_{50}$ values,[a] µg $ml^{-1}$: median (range)* Normal samples ($n = 11–13$) | Leukaemic samples ($n = 22–30$)[b] | *P-values[c]* Normal vs BM + PB ALL samples | Normal vs PB ALL samples |
|---|---|---|---|---|
| Prednisolone | 250 (1.58–>250) | 1.84 (<0.08–>250) | <0.05 | <0.05 |
| Dexamethasone | >0.8 (0.019–>0.8) | 0.029 (<0.0003–>0.8) | <0.01 | <0.01 |
| 6-Mercaptopurine | 410.7 (125–>500) | 125 (<15.6–>500) | <0.01 | <0.01 |
| 6-Thioguanine | 46.9 (20.2–>50) | 6.6 (2.7–>50) | <0.01 | <0.01 |
| Cytosine arabinoside | >2.5 (0.501–>2.5) | 0.469 (<0.002–>2.5) | <0.01 | <0.01 |
| Vincristine | 37.2 (8.2–>50) | 2.57 (<0.05–>50) | <0.01 | <0.01 |
| Vindesine | 41 (4.1–>50) | 2.6 (0.15–>50) | <0.01 | <0.01 |
| Daunorubicin | 1.112 (0.435–>2) | 0.092 (<0.002–>2) | <0.01 | <0.01 |
| Doxorubicin | >1 (0.48–>1) | 0.5 (0.08–>1) | <0.01 | <0.05 |
| Teniposide | 1.493 (0.77–6.58) | 0.263 (0.06–>8) | <0.01 | <0.01 |
| Mafosfamide | 16.2 (3.2–>100) | 6.6 (0.2–>100) | 0.16 | <0.05 |
| 4-Hydroperoxy-ifosfamide | not done | 5.4 (0.23–29.2) | | |
| L-Asparaginase[d] | >10 (1.26–>10) | 0.19 (<0.003–>10) | <0.01 | <0.01 |
| Mitoxantrone | 0.839 (0.14–>1) | 0.055 (0.001–>1) | <0.01 | <0.01 |

[a]Lethal concentration to 50% of the cells; [b]4-hydroperoxy-ifosfamide used in 13 samples; Paired samples: only PB samples included; [c]Wilcoxon's ranking test for unpaired data; [d]IU $ml^{-1}$.

PRD, DXM, 6-MP, 6-TG, VCR, DNR, Ara-C, VDS, Teni, L-Asp, Mitox and Dox.

We compared the range and median $LC_{50}$ values of each drug for all leukaemic BM samples and all leukaemic PB samples from 38 ALL patients. No significant differences were found between BM and PB leukaemic cells in sensitivity to any of the drugs (Table III). In 12 cases (11 ALL, 1 ANLL) drug sensitivity results from paired BM and PB samples were studied. Analysis of these paired data also showed no preferential sensitivity of BM or PB leukaemic cells to any of the drugs. Evaluating the individual data, 11 of the 12 patients showed no differences in drug sensitivity between their BM and PB leukaemic cells. One child with ALL showed a greater sensitivity of the BM cells. Although statistically significant, the actual differences in $LC_{50}$ values were small. There was a good correlation between the paired BM and PB $LC_{50}$ values in most individual cases and in the 106 paired BM and PB $LC_{50}$ comparisons together (Figure 3).

### Discussion

Several studies have compared the drug sensitivity of non-malignant and leukaemic cells. Table IV summarises the results of the studies in which patient samples were investigated. This table shows that in most studies non-malignant cells were found to be less sensitive to the drugs used than leukaemic cells. Occasionally, a greater sensitivity of the non-malignant cells was found (Scholzel *et al.*, 1986). For some drugs (e.g. DNR and Dox) the results are contradictory. This

**Table IV** Summary of relevant literature regarding *in vitro* drug sensitivity of human normal haemotopoetic cells *vs* human leukaemic cells

| *Reference* | *Cells* | *Assay* | *Drugs* | *Sensitive* *Less* | *Equally* (*compared to normal cells*) | *More* |
|---|---|---|---|---|---|---|
| Galili *et al.* (1980) | ALL, CLL, AML and CML *vs* normal BM and PB ly's | Viable cell count | Cortisol | | AML/CML | ALL/CLL |
| Spiro *et al.* (1981*a,b*) | CML-CFC *vs* normal BM- and PB-CFC | Clonogenic | Dox, DNR, L-Asp, Bs, Ara-C, HU, Mel, 6-TG m-AMSA | | CML (other drugs) | CML (AraC) |
| Speth *et al.* (1988) | ANLL BM *vs* normal BM progenitor | Clonogenic | Dox | | ANLL | |
| Singer *et al.* (1987) | AML-CFC (PB) *vs* normal GM-CFC | Clonogenic | Maf, Mel, Ara-C, 4-HC, VP-16 | | AML | |
| Schrek *et al.* (1967) | CLL (PB) *vs* normal PBL | Viable cell count | L-Asp | | | CLL |
| Schrek (1961, 1964) | CLL and LS *vs* normal PBL | Viable cell count | Cortisol, PRD | | | CLL/LS |
| Scholzel *et al.* (1986) | AML-CFC *vs* normal BM CFC-E and -M | Clonogenic | Mitox, DNR 4-dmDNR, 4'doDox | AML | | |
| Kluin-Nelmans *et al.* (1984) | L-CFC *vs* normal CFC-GM and BFU-E | Clonogenic | Maf | | L-CFC | |
| Jayaram *et al.* (1986) | ANLL and ALL *vs* normal BM leukocytes | Depression of [GTP] | Tiazofurin | | | ANLL/ALL |
| Greenberg *et al.* (1976) | L-CFC *vs* normal granulocytic CFC | Clonogenic | Ara-C, 6-TG | | L-CFC (6-TG) | L-CFC (AraC) |
| Buick *et al.* (1979) | AML-CFC *vs* normal granulopoetic and T-ly CFC | Colony | Dox, DNR | | | AML |
| Asselin *et al.* (1989) | ALL BM *vs* normal BM mononuclear | Viable cell count | L-Asp | | | ALL |
| Weisenthal *et al.* (1987) | ALL and CLL *vs* normal PBL | DiSC | VCR | | | ALL/CLL |
| Taetle *et al.* (1983) | (B-)CLL-CFC *vs* normal T-PBL | Clonogenic | HC, 5-FU, Mel, MTX, Dox, Blm, CA, CP, Ara-C | | CLL (Blm) | CLL (other drugs) |
| | | | | | MTX not evaluable | |
| Werthamer *et al.* (1971) | CLL *vs* normal PB ly's | RNA and protein precursor incorporation | Cortisol | | | CLL |
| Verdonck *et al.* (1990) | AML-, ALL- and CMF-CFC *vs* normal BM CFC-GM and -GEMM | Clonogenic | alkyllysophospholipid | | | AML/ALL/CML |
| Katano *et al.* (1989) | ALL BM *vs* normal BM mononculear | Bromodeoxy- uridine incorporation in S-phase cells | Ara-C | | ALL | |
| Potter *et al.* (1980) | AML BM *vs* normal BM | $^3$H Thymidine incorporation | Ara-C | | | AML |

AML: acute myeloid leukaemia; BFU-E: blood forming units-erythroid; Blm: bleomycine; Bs: busulfan; CA: chlorambucil; CFC: colony forming cells; CFC-E: CFC-erythroid; CFC-GEMM: CFC-granulocyte-erythrocyte-macrophage-megakaryocyte; CFC-GM: CFC-granulocytic-myeloid; CFC-M: CFC-myeloid; CLL: chronic lymphocytic leukaemia; CML: chronic myeloid leukaemia; CP: cisplatinum; 4-dmDNR: 4-demethyoxy DNR; 4'doDox: 4'deoxyDox; 5-FU: 5-fluorouracil; GTP: guanyl triphosphate; HC: hydrocortisone; 4-HC: 4-hydroperoxycyclofosfamide; HU: Hydroxy-ureum; L-CFC: leukaemic-CFC; LS: lymphosarcoma in leukaemic phase; ly: lymphocyte; Mel: melphalan; MTX: methotrexate. Other abbreviations: See Table I and text.

**Figure 3** Comparison of *in vitro* drug sensitivity between (paired) BM and PB leukaemic cells from 12 patients. Each point represents a paired $LC_{50}$ value ($n = 106$). The line shown is the line x = y.

might be explained by the differences in assays, drug concentrations and cells used.

We demonstrated, using the MTT assay, that normal PB lymphocytes are significantly more resistant to PRD, DXM, 6-MP, 6-TG, VCR, DNR, Ara-C, VDS, Teni, L-Asp, Mitox and Dox than leukaemic cells of children with ALL, also when only leukaemic samples from the PB were evaluated (Table II). We did not find a significantly greater sensitivity of the BM and PB leukaemic cells to Maf (an active metabolite of CFM), in accordance with the results of Kluin-Nelemans *et al.* (1984) and Singer and Linch (1987). In two out of three studies using melphalan, another alkylating agent, again an equal sensitivity of non-malignant and leukaemic cells was reported (Spiro *et al.*, 1981*a*; Singer & Linch, 1987). These observations suggest that alkylating agents have a less favourable therapeutic index. Their value in BM purging – based on direct cytotoxic activity – seems to be small. This does not necessarily implicate that these drugs are not useful in this respect. Recently, Rizzoli *et al.* (1990) reported a beneficial effect of BM puring with Maf (in individually adjusted doses) on leukaemic free survival after autologous-purged – BM transplantation and suggested this could be due to activation of immunological systems able to control minimal residual disease *in vivo*, and not primarily to the direct cytotoxic activity of the drug during the purging procedure. The wide range of drug sensitivity of leukaemic cells from different patients, implies the necessity of individually adjusted doses of cytostatic drugs for optimal BM purging, as indeed has been reported to be more successful than standard purging (Rizzoli *et al.*, 1990).

The cause(s) of the presented differential drug sensitivity of normal lymphocytes and leukaemic cells are largely unknown. However, these cells differ in several aspects, like immunophenotype and differentiation-stage. Normal PB lymphocytes are mainly of the T-lineage (which was even more accentuated after the 4-days incubation period of the MTT assay in the present study) and represent mature cells. Most childhood actue leukaemias are of the immature B-lineage phenotype, as was the case in this study. The T-lineage leukaemias show a less mature phenotype than normal T-cells. Recently we found that T-ALL cells were relatively resistant to various drugs compared to immature B-ALL cells (Pieters *et al.*, in press). Therefore, the differences regarding immunophenotype between the tested leukaemic and normal lymphocytes could well contribute to the presented differences in drug sensitivity. Changes in immunophenotype of normal lymphocytes might occur in patients suffering a malignancy. However, we found that the removal of normal T-lymphocytes from ALL samples with less than 80% ALL cells resulted in increased drug sensitivity (unpublished data). This supports the conclusions of the present study. An extensive discussion of all other possible causes of the presented difference in drug sensitivity is beyond the scope of this report.

The analysis of the assay results obtained with (unpaired) BM and PB leukaemic cells from all 38 ALL patients did not reveal a preferential sensitivity of the PB or BM leukaemic cells to any of the drugs (Table III). Similarly, no preferential sensitivity of the (paired) BM or PB leukaemic cells from 12 leukaemic patients (one ANLL, 11 ALL) was found. In 11 out of 12 patients of whom we tested their (paired) BM and PB leukaemic cells, the sample source did not significantly influence drug sensitivity. In one case the leukaemic cells from the BM were significantly more sensitive than those from the PB, but the actual differences in $LC_{50}$ values were very small and of no practical importance. The correlation for all 106 paired BM and PB $LC_{50}$ comparisons was very good, with most pairs close to the ideal line x = y (Figure 3). Our findings agree with those of Bird *et al.* (1986), who reported a significant association in sensitivity to a maximum of six drugs of leukaemic BM and PB cells from 12 patients. The same was found by Sargent and Taylor (1989) and Spiro *et al.* (1981*a*), in single cases.

We conclude that normal PB lymphocytes are more resistant than childhood ALL cells to a large number of drugs *in vitro*. Consequently, the number of normal lymphocytes contaminating an ALL sample to be tested should be low, when the MTT assay or a similar total cell kill assay is used. This is especially the case in view of the higher survival at day 4 and the higher MTT reduction per living cell of the normal lymphocytes compared to the untreated ALL cells. The MTT assay and the Differential Staining Cytotoxicity (DiSC) assay gave comparable results in samples with 80% (or more) leukaemic cells, the lowest percentage tested (Pieter *et al.*, 1989). Therefore, in case of a sample with less than 80% leukaemic cells, the use of the DiSC assay – in which a distinction between non-malignant and leukaemic cells can be made – should be considered. Because this assay is laborious and subjective, we are investigating techniques to remove non-malignant cells from leukaemic samples.

Finally, because the drug sensitivity profiles of leukaemic cells from the BM are quite comparable to those from the PB, it is allowed to evaluate results obtained using samples from both sources together, which obviously is of practical importance.

The laboratory of the Dutch Childhood Leukemia Study Group (DCLSG) provided most of the patient samples. Board members of the DCLSG are J.P.M. Bökkerink, M.V.A. Bruin, P.J. Van Dijken, K. Hählen, W.A., Kamps, E.F. Van Leeuwen, F.A.E. Nabben, A. Postma, J.A. Rammeloo, I., Risseeuw-Appel, G.A.M. De Vaan, E. Th. Van't Veer-Korthof, A.J.P. Veerman, F.C. De Waal, M. Van Weel-Sipman and R.S. Weening.

### References

ASSELIN, B.L., RYAN, D., FRANTZ, C.N. & 4 others (1989). In vitro and *in vivo* killing of acute lymphoblastic leukaemia cells by L-asparaginase. *Cancer Res.*, **49**, 4363.

BIRD, M.C., FORSKITT, S., GILBY, E.D. & BOSANQUET, A.G. (1986). The influence of sample source and cell concentration on the *in vitro* chemosensitivity of haematological tumours. *Br. J. Cancer*, **53**, 539.

BLACK, M.M. & SPEER, F.D. (1953). Effects of cancer chemotherapeutic agents on dehydrogenase activity of human cancer tissue *in vitro*. *Am J. Clin. Pathol.*, **23**, 218.

BUICK, R.N., MESSNER, H.A., TILL, J.E. & MCCULLOCH, E.A. (1979). Cytotoxicity of adriamycin and daunorubicin for normal and leukemia progenitor cells of man. *J. Natl Cancer Inst.*, **62**, 249.

GALILI, U., PROKOCIMER, M. & IZAK, G. (1980). The *in vitro* sensitivity of leukemic and normal leukocytes to hydrocortisone induced cytolysis. *Blood*, **56**, 1077.

GREENBERG, P.L., VAN KERSEN, I. & MOSNY, S. (1976). Cytotoxic effects of 1-beta-D-arabinofuranosylcytosine and 6-thioguanine in vitro on granulocytic progenitor cells. *Cancer Res.*, **36**, 4412.

JAYARAM, H.N., PILLWEIN, K., NICHOLS, C.R., HOFFMAN, R. & WEBER G. (1986). Selective sensitivity to tiazofurin of human leukemic cells. *Biochem. Pharmacol.*, **35**, 2029.

KATANO, N., TSURUSAWA, M., NIWA, M. & FUJIMOTO, T. (1989). Flow cytometric determination with bromodeoxyuridine/DNA assay of S-phase cells to cytosine arabinoside in chidhood acute lymphoblastic leukemia. *Am. J. Pediatr. Hematol./Oncol.*, **11**, 411.

KIRKPATRICK, D.L., DUKE, M. & GOH, T.S. (1990). Chemosensitivity testing of fresh human leukaemia cells using both a dye exclusion assay and a tetrazolium dye (MTT) assay. *Leuk. Res.*, **14**, 459.

KLUIN-NELEMANS, H.C., MARTENS, A.C., LÖWENBERG, B. & HAGENBEEK, A. (1984). No preferential sensitivity of clonogenic AML cells to ASTA-Z-7557. *Leuk. Res.*, **8**, 723.

MOSMANN, T. (1983). Rapid colorimetric assay for cellular growth and survival: application to proliferation and cytotoxicity assays. *J. Immunol. Methods*, **65**, 55.

PIETERS, R., HUISMANS, D.R., LEYVA, A. & VEERMAN, A.J.P. (1988). Adaptation of the rapid automated tetrazolium dye based (MTT) assay for chemosensitivity testing in childhood leukemia. *Cancer Lett.*, **41**, 323.

PIETERS, R., HUISMANS, D.R., LEYVA, A. & VEERMAN, A.J.P. (1989). Comparison of the rapid automated MTT-assay with a dye exclusion assay for chemosensitivity testing in childhood leukaemia. *Br. J. Cancer*, **59**, 217.

PIETERS, R., LOONEN, A.H., HUISMANS, D.R. & 5 others (1990). *In vitro* sensitivity of cells from children with leukemia using the MTT assay with improved culture conditions. *Blood*, **76**, 2327.

PIETERS, R., KASPERS, G.J.L., VAN WERING, E.R. & 4 others (1991). *In vitro* drug resistance in childhood acute lymphoblastic leukemia in relation to age and immunophenotype. *Haematol. Blood Transf.* (in press).

POTTER, C.G. & BUNCH, C. (1980). Sensitivity of normal and acute myelogenous leukaemia marrow cells to inhibition by cytosine arabinoside. *Br. J. Cancer*, **41**, 985.

RIZZOLI, V., CARELLA, A.M., CARLO-STELLA, C. & MANGONI, L. (1990). Autologous marrow transplantation in acute lymphoblastic leukemia: control of residual disease with mafosfamide and induction of syngeneic GVHD with cyclosporin. *Bone Marrow Transplant.*, **6** (suppl 1), 76.

SARGENT, J.M. & TAYLOR, C.G. (1989). Appraisal of the MTT assay as a rapid test of chemosensitivity in acute myeloid leukaemia. *Br. J. Cancer*, **55**, 595.

SCHOLZEL, C., VAN PUTTEN, W. & LOWENBERG, B. (1986). A comparison of *in vitro* sensitivity of acute myeloid leukemia precursors to mitixantrone, 4'deoxydoxorubicin, 4-demethoxydaunorubicin and daunorubicin. *Leuk. Res.*, **10**, 1455.

SCHREK, R. (1961). Cytotoxicity of adrenal cortex hormones on normal and malignant lymphocytes of man and rat. *Proc. Soc. Exp. Biol. Med.*, **108**, 328.

SCHREK, R. (1964). Prednisolone sensitivity and cytology of viable lymphocytes as tests for chronic lymphocytic leukemia. *J. Natl Cancer Inst.*, **33**, 837.

SCHREK, R., DOLOWY, W.C. & AMMERAAL, R.N. (1967). L-asparaginase: toxicity to normal and leukemic man lymphocytes. *Science*, **155**, 329.

SINGER, C.R.J. & LINCH, D.C. (1987). Comparison of the sensitivity of normal and leukaemic myeloid progenitors to *in vitro* incubation with cytotoxic drugs: a study of pharmacological purging. *Leuk. Res.*, **11**, 953.

SPETH, P.A.J., RAIJMAKERS, R.A.P., BOEZEMAN, J.B.M. & 4 others (1988). *In vitro* cellular adriamycin concentrations related to growth inhibiton of normal and leukemic human bone marrow cells. *Eur. J. Cancer Clin. Oncol.*, **24**, 667.

SPIRO, T.E., MATTELAER, M.A., EFIRA, A. & STRUYCKMANS, P. (1981*a*). Sensitivity of myeloid progenitor cells in healthy subjects and patients with chronic myeloid leukemia to chemotherapeutic agents. *J. Natl Cancer Inst.*, **66**, 1053.

SPIRO, T.E., SOCQUET, M., DELFORGE, A. & STRUYCKMAN, P. (1981*b*). Chemotherapeutic sensitivity of normal and leukemic hematopoietic progenitor cells to N-[4-(9-acridinylamino)-3-methoxyphenyl]-methanesulfonamide, a new anticancer agent. *J. Natl Cancer Inst.*, **66**, 615.

TAETLE, R., TO, D. & MENDELSOHN, J. (1983). *In vitro* sensitivity to steroid hormones and cytotoxic agents of normal and malignant lymphocyte colony-forming cells. *Cancer Res.*, **43**, 3553.

VEERMAN, A.J.P. & PIETERS, R. (1990). Drug sensitivity assays in leukaemia and lymphoma. *Br. J. Haematol.*, **74**, 381.

VEERMAN, A.J.P., HUISMANS, D.R. & VAN ZANTWIJK, C.H. (1983). Immunological phenotype related to acid alpha-naphthyl-acetate-esterase and acid phosphatase in childhood acute lymphoblastic leukaemia. *Acta Haematol.*, **69**, 2.

VERDONCK, L.F., WITTEVEEN, E.O., VAN HEUGTEN, H.G., ROZEMULLER, E. & RIJKSEN, G. (1990). Selective killing of malignant cells from leukemic patients by alkyl-lysophospholipid. *Cancer Res.*, **50**, 4020.

WEISENTHAL, L.M., SU, Y.-Z., DUARTE, T.E., DILL, P.L. & NAGOURNEY, R.A. (1987). Perturbation of *in vitro* drug resistance in human lymphatic neoplasms by combinations of putative inhibitors of protein kinase C. *Cancer Treat. Rep.*, **71**, 1239.

WERTHAMER, S. & AMARAL, L. (1971). The response of leukemic lymphocytes to cortisol: a suggested role of transcortin. *Blood*, **37**, 463.